# The genetic lottery goes to school: Better schools compensate for the effects of students’ genetic differences

**DOI:** 10.1073/pnas.2511715122

**Published:** 2025-10-24

**Authors:** Rosa Cheesman, Nicolai Borgen, Astrid M. J. Sandsør, Paul Hufe

**Affiliations:** ^a^The PROMENTA Center, Department of Psychology, University of Oslo, Oslo 0317, Norway; ^b^PsychGen Center, Norwegian Institute of Public Health, Oslo 0473, Norway; ^c^Center for Research on Equality in Education (CREATE), Oslo 0318, Norway; ^d^Department of Special Needs Education, University of Oslo, Oslo 0318, Norway; ^e^School of Economics, University of Bristol, Bristol BS8 1TU, United Kingdom

**Keywords:** education, gene–environment interaction, polygenic indices, school value-added

## Abstract

In this paper, we investigate whether better schools can compensate for the effects of children’s genetic differences. To this end, we combine data from the Norwegian Mother, Father, and Child Cohort Study (MoBa) with Norwegian register data to estimate the interaction between measures of children’s predisposition to education and school quality. We use MoBa’s genetic data to compute polygenic indices for educational attainment (*PGI^EA^*). Importantly, MoBa includes genetic data on mother-father-child trios, allowing us to identify causal genetic effects using within-family variation. We calculate school value-added measures from Norwegian register data, allowing us to causally estimate school quality effects. Leveraging the advantages of both data sources, we provide a causally identified study of gene–environment interactions in the school context. We find evidence for substitutability of *PGI^EA^* and school quality in reading but not numeracy: A 1 SD increase of school quality decreases the impact of a 1 SD increase of *PGI^EA^* on reading test scores by 6%. The substitutability arises through gains of students at the lower end of the *PGI^EA^* distribution. This suggests that investments in school quality may help reduce educational inequalities arising from genetic differences between students.

## Significance

Education is a core determinant of life outcomes, and equity in educational systems is a central policy goal. An important question in the literature is whether schools can reduce inequities arising from social background and genetic differences between children. Using causal estimates of gene–environment interactions in the school context, we investigate whether schools can compensate for genetic differences captured by polygenic indices for educational attainment. We find a negative gene–environment interaction for reading skills but not numeracy, indicating that schools can compensate for the effects of differences in polygenic indices for educational attainment.

A core topic in the social sciences is the question of how well schools reduce the inequity of birth. A substantial body of literature has explored the extent to which schools fulfill their purpose of compensating for the effects of background differences among children that lie beyond their control. Traditionally, this research has focused on inequalities in school performance by socioeconomic status (SES), race, and gender (1–3). Here, we consider whether schools compensate or reinforce the effects of children’s genetic differences.

Genetic differences between children play a significant role in skills development, with the twin study heritability for childhood school performance estimated at approximately 50% (4, 5). Notably, these genetic influences interact with social influences on academic skills, such as family socioeconomic status (6) and the broader sociopolitical environment (7). Evidence of gene–environment interactions is highly relevant to social policy; it underscores the fact that environments do not affect all individuals equally. Interventions that counteract genetic influences could serve as vital policy levers for fostering educational equity.

Given the potential policy implications, understanding gene–environment interactions in educational settings is of particular interest. Traditional behavioral genetic study samples limit research on genetic interactions with school environments because twins usually attend the same school. Nonetheless, several twin studies have examined gene–environment interactions within schools. Taylor et al. (8) found that genetic variance in oral reading fluency increased with higher teacher quality (as measured by classroom reading gains). Guimond et al. (9) found that genetic effects on academic achievement were stronger when teachers used frequent praise and infrequent punishment—similarly supporting genetic amplification rather than compensation in more advantaged educational settings.

The recent availability of polygenic indices (PGIs) has catalyzed research into how genetic factors interplay with school environments. While twin studies capture total heritability, PGIs capture only a subset of genetic effects—those indexed by variants identified in genome-wide association (GWA) studies, such as of educational attainment. In contrast to twin studies, PGIs estimate genetic effects at the individual level, allowing for analyses based on broader and more representative population samples. Importantly, the genetic associations identified in GWA studies of educational outcomes reflect effects that emerge within the particular normative social and educational contexts of study, rather than representing inherent genetic disadvantages. The variants associated with lower educational attainment might be different in different contexts.

The emerging PGI-based literature reveals complex patterns of gene-by-school environment interactions. On one hand, Harden et al. (10) found that schools with greater socioeconomic resources appeared to compensate for genetic disadvantages in reducing dropout from basic math while reinforcing genetic advantages in the pursuit of advanced mathematics. Two other school-level studies yielded inconclusive or null results, finding that initial gene–environment interactions disappear when accounting for scale artifacts and school socioeconomic composition (11, 12). On the other hand, two recent studies have found that higher-quality schools compensate for the effects of differences in educational attainment PGIs on test scores and years of schooling (13, 14).

These varying findings suggest that gene–environment interaction findings in education research may depend on various factors including: 1) the specific educational outcome examined—different patterns may emerge for basic skill acquisition (such as decoding in reading) versus advanced achievement (such as complex problem-solving); 2) the developmental stage being studied, since genetic and environmental influences may shift from elementary through high school and beyond; 3) subject matter, as numeracy and reading may each show distinct patterns of heritability and environmental sensitivity; and 4) crucially, whether studies measure school quality in a way that captures schools’ effectiveness to promote learning rather than simply reflecting the predetermined characteristics of their student populations (e.g., student ability, parental socioeconomic status, and ethnicity).

The primary challenge in existing research is the difficulty of identifying exogenous variation in both genetic factors and school quality measures. Estimates of gene–environment interactions (GxE) are biased when genetic factors (G) and/or the environment of interest (E) correlate with other variables that influence outcomes. An effective approach to address the first source of bias is by using within-family PGIs. Within-family variation in PGIs is a random “genetic lottery” (15), meaning that effects cannot be confounded by other family characteristics. This approach was first adopted by Cheesman et al. (16) in the context of gene–environment interactions within schools. However, the study lacked a method for capturing exogenous variation in school quality and was limited in capturing only the school environments of children participating in the cohort study. Without identifying causal effects, we cannot conclude whether social advantages increase the expression of genetic differences or instead support compensation and diathesis stress models of child development, whereby social advantages buffer genetic influences.

Here, we address these concerns by incorporating exogenous variation in both genetics and environments. We leverage within-family PGIs through parent–offspring genetic data in the Norwegian Mother Father Child Cohort study (MoBa) and use established school value-added (VA) indicators, integrating data from Norwegian registers, thus encompassing all Norwegian children in the cohort (17, 18). The Norwegian context and rich educational data provide the ideal setting for validating our causal gene–environment interaction approach.

## Results

### Analysis Sample.

Our analysis is based on the Norwegian Mother, Father, and Child Cohort Study (MoBa) (19), linked to population-wide administrative records containing students’ national standardized test scores and school identifiers. The outcomes of interest are students’ reading and numeracy test scores in grade 9, which capture students’ skills in these domains 1 y after starting lower secondary school (grades 8-10; see *SI Appendix*, sections A and B, for details on the schooling system and national test scores in Norway). Students’ test scores are standardized within each year in the full population of Norwegian students to have a mean of zero and a SD of one. We measure students’ and their parents’ genetic predisposition to educational attainment with a polygenic index for educational attainment *PGI^EA^* and quantify school quality through school VA measures (*VA^d^* with *d* ∈ {reading, numeracy}). *VA^d^* is constructed using the full population of Norwegian students. For our main analysis, we standardize *PGI^EA^* and *VA^d^* in our estimation sample to have a mean of zero and a SD of one.

Table 1 shows descriptive statistics for our analysis sample comprising 30,939 children with complete data on all relevant outcomes, treatment, and control variables. While our analysis sample of genotyped parent–child trios is comparable to the overall MoBa sample, MoBa participants are positively selected on socioeconomic background characteristics and are therefore not fully representative of the overall Norwegian population. However, this selection does not compromise the internal validity of our estimates or their causal interpretation.

**Table 1.** Summary statistics

	Analysis sample *N* = 30,939				MoBa (All) *N* = 56,533		Population *N* = 331,591	
Variable	Mean	SD	Min	Max	Mean	SD	Mean	SD
Birth year	2004.9	1.6	2002	2008	2004.8	1.6	2004.5	1.7
Female	0.5	0.5	0.0	1.0	0.5	0.5	0.5	0.5
Migration background	0.1	0.3	0.0	1.0	0.1	0.3	0.2	0.4
Education (Father)	14.6	2.6	7.0	21.0	14.4	2.7	13.7	2.9
Education (Mother)	15.1	2.3	9.0	21.0	15.0	2.4	14.1	2.9
Inc. rank (Father)	58.5	25.6	0.0	99.0	57.1	26.2	50.9	28.3
Inc. rank (Mother)	61.0	25.4	0.0	99.0	59.9	25.7	51.5	27.6
Age (Father)	32.9	5.1	18.0	65.0	33.1	5.3	33.2	6.0
Age (Mother)	30.5	4.4	16.0	47.0	30.6	4.5	30.2	5.1
Reading (Grade 8)	0.3	0.9	-3.2	2.4	0.2	0.9	0.1	1.0
Numeracy (Grade 8)	0.3	0.9	-2.5	2.5	0.2	1.0	0.0	1.0
English (Grade 8)	0.2	1.0	-2.4	2.2	0.1	1.0	0.0	1.0

This table shows descriptive statistics. The first panel focuses on the main analysis sample, i.e., MoBa cohorts 2002-2007 with *PGI^EA^* data for biological mothers, fathers, and their children. The second panel also includes MoBa participants with missing *PGI^EA^* data for either mothers, fathers, or their children. The third panel focuses on the entire Norwegian population born 2002-2007, irrespective of whether they have participated in MoBa. Parental income ranks are calculated in the full population. Test scores for reading, numeracy, and English are standardized on the full population. Data: Own calculations based on MoBa and Norwegian registers. Note that, while 2007 was the latest complete birth cohort available during data construction, a small number of children born in 2008 (n = 13) also met all inclusion criteria and were included in the analytical sample.

### Validation of Identification Assumptions.

#### Exogenous variation in polygenic indices (*PGI^EA^*).

Causal identification of gene–environment interactions requires exogenous variation in both genetic factors and environmental exposures (20). In the absence of exogenous variation in children’s education-linked genetics (*PGI^EA^*), estimates of genetic effects and the corresponding gene–environment interaction will be confounded by indirect genetic effects from parents and population stratification (21). We achieve exogenous variation in *PGI^EA^* by leveraging the availability of genetic trios in MoBa. By analyzing children’s *PGI^EA^* while controlling for maternal and paternal *PGI^EA^*, we isolate the component of children’s genetic variation that is randomly allocated during meiosis. This within-family genetic variation approach enables causal identification of genetic effects (22).

**Fig. 1.**
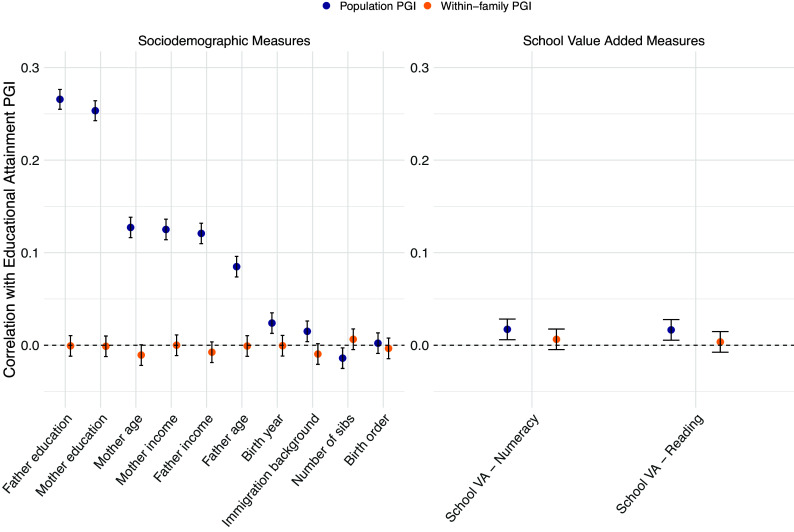
Validation of the exogeneity of within-family *PGI^EA^* variation. This plot shows correlations of *PGI^EA^* with children’s observable sociodemographic background characteristics and school value-added (*VA^d^*) in our analysis sample (*N* = 30,939). Dark blue circles show uncontrolled population-level correlations with children’s *PGI^EA^.* Orange circles show the corresponding within-family correlations after controlling for *PGI^EA^* of mothers and fathers. Whiskers show 95% CI. SE are heteroskedasticity robust. Data: Own calculations based on MoBa and Norwegian registers.

Fig. 1 provides evidence supporting the exogeneity of the within-family *PGI^EA^* variation used in our study. Specifically, it shows correlations between students’ *PGI^EA^* and family characteristics that may influence the educational outcomes of children, such as parents’ education and income. The dark blue dots show correlations without controls for parental *PGI^EA^*. Many of the correlations are positive and significantly different from zero—a pattern consistent with indirect parental genetic effects. The orange dots show the same correlations after controlling for parental *PGI^EA^*. Notably, all correlations with family characteristics converge to zero and become statistically indistinguishable from zero. In line with our expectations, the residual within-family genetic variation of *PGI^EA^* is not confounded with other family characteristics that may influence children’s educational outcomes, suggesting a causal interpretation of our estimated genetic effects. Fig. 1 also demonstrates that *PGI^EA^* and *VA^d^* are not correlated with each other after conditioning on parental *PGI^EA^*. The absence of such gene–environment correlations suggests that we have sufficient independent variation in *PGI^EA^* and *VA^d^* to separately identify genetic effects, school effects, and the gene–environment interaction of interest.

#### Exogenous variation in school VA.

**Fig. 2.**
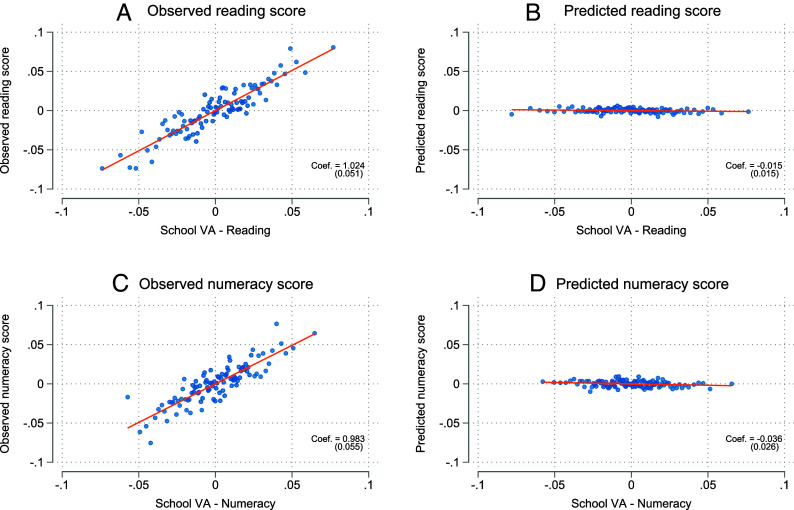
Validation of the exogeneity of *VA^d^.* This figure shows the association between *VA^d^* and children’s observable and predicted test scores in reading (Panels [*A*] and [*B*]) and numeracy (Panels [*C*] and [*D*]) in grade 9 for the full population of cohorts 1997-2007 (*N* = 508,615). All variables are residualized from control variables *Z* (*Materials and Methods*). *VA^d^* is reported in raw scores, i.e., values that have not been standardized. Predicted test scores are constructed from children’s reading and numeracy test scores in grade 5, maternal and paternal earnings rank at age 6, and maternal and paternal age at birth. Scatter plots are constructed by binning the *VA^d^* distribution into 100 percentiles. Regression slopes are estimated on the full data. SE are clustered at the school level. Data: Own calculations based on Norwegian registers.

VA models estimate the causal effect of schools on student outcomes by comparing students’ academic progress relative to comparable peers at different schools (see Angrist et al. (17) for a recent overview article). The core identification challenge is to isolate *VA^d^* from other factors that contribute to student outcomes. For example, it is well documented that school enrollment is not random but stratified by factors such as student ability, parental socioeconomic status, and ethnicity (3, 23, 24). As these factors contribute to student outcomes, uncontrolled comparisons of educational outcomes across schools will yield biased estimates of *VA^d^*. In some settings, researchers can exploit random student assignments based on lotteries to estimate *VA^d^* net of confounding factors (3, 25). In the absence of random assignment, however, we can mimic such experimental variation using observational data following the protocols suggested by Chetty et al. (26) and Jackson et al. (2). In particular, we calculate *VA^d^* while conditioning on observable differences across students, including differences in family socioeconomic status and prior student test scores. Therefore, the identification of school effects relies on the assumption that the predetermined characteristics are sufficient to control for selection into schools. Existing literature has documented that the inclusion of prior test scores usually satisfies this assumption (2, 26). Fig. 2 provides evidence supporting the predictive validity and the exogeneity of *VA^d^* in our study. In Panel (a), we assess whether our measure of school *VA^d^* captures relevant variation in student outcomes. To this end, we regress the outcome of interest, i.e., student’s reading test scores in grade 9, on the corresponding *VA^d^* measure of their school. The slope is precisely estimated and cannot be statistically distinguished from one: A one-unit increase in *VA^d^*, on average, increases reading test scores by one unit as well. In the VA literature, this property is often called “forecast unbiasedness” and establishes the high predictive validity of *VA^d^* for the corresponding student outcome (17, 26).

In Panel (*B*), we assess whether this relationship is potentially confounded by selection based on unobserved characteristics. To that end, we predict student test scores from a variety of variables that we do not control for in the construction of *VA^d^* and regress these predicted test scores on *VA^d^*. A coefficient different from zero would suggest that unobserved variables determine selection into schools, and we would have to reject the exogeneity of *VA^d^*. However, the slope is flat and very close to zero: A one-unit increase in school *VA^d^* decreases predicted reading test scores by 0.015 SD. Similar to findings in other studies (2, 18, 26), this suggests that there is negligible bias in our VA estimates after conditioning on a set of controls, including students’ prior test scores. In Panels (*C*) and (*D*), we repeat the same exercise for numeracy test scores. The results are almost identical, further supporting our identification assumption.

#### Metric properties of outcomes.

Several measurement problems can create spurious gene–environment interactions, e.g., test scores having ceiling effects; restricted range of test scores at higher *VA^d^* levels, or curved relationships being misspecified as linear (20, 27–29). While the raw test scores show some negative skew (*SI Appendix*, Fig. S2), the standardized test scores used in our analyses do not display problematic skew or ceiling effects. We find that test scores display only slightly lower variance at higher school quality levels (*SI Appendix*, Fig. S3), and that relationships between key variables of interest are linear (*SI Appendix*, Fig. S4).

### Gene–environment interaction.

#### Reading.

Table 2 documents that more effective schools have higher relative impacts on the reading outcomes of children with a low *PGI^EA^*.

**Table 2.** Gene–environment interaction for reading and numeracy test scores

	(1)	(2)	(3)	(4)
Panel (a): Reading (Grade 9)				
*PGI^EA^*	0.304*** (0.006)	0.230*** (0.008)	0.231*** (0.005)	0.231*** (0.005)
*VA^d^*	0.091*** (0.014)	0.090*** (0.013)	0.052*** (0.007)	0.050*** (0.007)
*PGI^EA^* × *VA^d^*	−0.020* (0.008)	−0.020* (0.008)	−0.013* (0.005)	−0.013 (0.007)
Genetic controls	×	×	✓	✓
School quality controls	×	×	✓	✓
2-way interactions (*PGI^EA^*, *VA^d^*, *X*)	×	×	×	✓
R^2^	0.096	0.104	0.654	0.657
N	30,939	30,939	30,939	30,939
Skill persistence ρ	–	–	0.462*** (0.006)	0.460*** (0.006)
Panel (b): Numeracy (Grade 9)				
*PGI^EA^*	0.314*** (0.006)	0.238*** (0.008)	0.239*** (0.004)	0.239*** (0.004)
*VA^d^*	0.076*** (0.013)	0.075*** (0.013)	0.039*** (0.005)	0.040*** (0.005)
*PGI^EA^* × *VA^d^*	−0.005 (0.007)	−0.006 (0.007)	−0.000 (0.004)	0.001 (0.005)
Genetic controls	×	×	✓	✓
School quality controls	×	×	✓	✓
2-way interactions (*PGI^EA^*, *VA^d^*, *X*)	×	×	×	✓
R^2^	0.102	0.109	0.738	0.740
N	30,939	30,939	30,939	30,939
Skill persistence ρ	–	–	0.702*** (0.004)	0.703*** (0.004)

This table shows estimates for the effects of *PGI^EA^* and *VA^d^* on children’s reading scores (Panel [a]) and numeracy scores (Panel [b]) in grade 9, as well as the corresponding gene–environment interaction. Genetic controls include the *PGI^EA^* of biological mothers and fathers, and categorical variables for the genotyping batch. School quality controls include lagged grade 8 test scores in reading, numeracy, English, maternal and paternal years of education, second-generation migration status, gender, birth cohort, birth order, number of siblings, and school-cohort averages of all previous controls. Two-way interactions include all interactions of *PGI^EA^* and *VA^d^* with the aforementioned controls. Skill persistence ρ indicates the estimate for lagged test scores in reading/numeracy (grade 8), which is estimated in the model as part of the child controls. SE (in parentheses) are clustered at the school level. Significance levels: *P < 0.05, **P < 0.01, ***P < 0.001. Data: Own calculations based on MoBa and Norwegian registers.

In our base model without any controls (column 1), a 1 SD increase in children’s *PGI^EA^* is associated with 0.304 SD higher reading scores, while a 1 SD increase in *VA^d^* is associated with 0.091 SD higher reading scores. The interaction between *PGI^EA^* and *VA^d^* is negative and significant at the 5% level, suggesting that the effect of children’s genetic predispositions captured by their *PGI^EA^* on their reading skills is moderated by school quality. However, these estimates lack a causal interpretation due to potential confounding by indirect genetic effects and nonrandom selection into schools.

In column (2), we incorporate controls for parental *PGI^EA^* and genotyping protocols. In this model, a 1 SD increase in *PGI^EA^* increases reading test scores by 0.230 SD. The drop in the effect of *PGI^EA^* in comparison to column (1) is consistent with established literature that suggests that 40 to 50% of the raw *PGI^EA^* associations with academic skills reflect indirect genetic effects and population stratification (21, 30, 31). After controlling for *PGI^EA^* of parents, our estimates rely on random within-family variation only and are not confounded by other family characteristics that may correlate with educational outcomes. The effect of *PGI^EA^* has a causal interpretation.

In column (3), we incorporate the full set of controls used in *VA^d^* construction, including lagged test scores and school-cohort characteristics (17). The effect of *PGI^EA^* remains stable, highlighting its causal interpretation after accounting for the *PGI^EA^* of parents. However, the effect of *VA^d^* drops from 0.091 to 0.052. This drop is expected since controlling for parental *PGI^EA^* is insufficient to control for selection into schools. After accounting for the expanded set of covariates, our estimates of school effects account for selection into schools and are not confounded by other family characteristics that may correlate with educational outcomes. The effect of *VA^d^* has a causal interpretation.

In column (3), we can give the base effects of both *PGI^EA^* and *VA^d^* a causal interpretation. However, following the arguments of Keller (32) and Feigenberg et al. (33), it is still an open question whether we can give the gene–environment interaction a causal interpretation as well. Since our treatment variables are considered exogenous conditional on a set of covariates, we need to include the full set of two-way interactions of these covariates with the variables of interest (*PGI^EA^, VA^d^*) to ensure that the gene–environment interaction is not picking up spurious correlations. Importantly, however, when including these two-way interactions, the researcher faces a bias–variance tradeoff. On the one hand, the integration of two-way interactions is necessary for the unbiased estimation of the gene–environment interaction if these two-way interactions are correlated with the outcome and the gene–environment interaction of interest (32, 33). On the other hand, the two-way interactions may lead to a loss of statistical power and inflate SE, particularly when degrees of freedom decrease substantially, *R^2^* increases minimally, or collinearity exists between interaction terms (33, 34).

In column (4), we augment our regression model by adding all two-way interactions of *PGI^EA^* and *VA^d^* with the vector of covariates *X*. If our gene–environment interaction of interest was confounded by other interactions between our variables of interest and the controls, we would expect the point estimate of *PGI^EA^ *×* VA^d^* in column (4) to diverge from the corresponding estimate in column (3). However, this is not the case. The estimate of the gene–environment interaction remains stable, but the SE increase from 0.005 to 0.007. This suggests that the magnitude of the interaction between the *PGI^EA^* and *VA^d^* identified in column (3) is unbiased but becomes nonsignificant in column (4) due to the increased variance of the estimates. In view of the stability of the point estimates and in line with the arguments put forward by Feigenberg et al. (33), we focus on the estimates in column (3) as our preferred estimates.

Our preferred estimate for the gene–environment interaction in column (3) suggests that a 1 SD increase in *PGI^EA^* increases the reading test scores of students in the average school in Norway by 0.231 SD. For students attending a school 1 SD above the country average, the impact of a 1 SD increase in *PGI^EA^* decreases by approximately 6% [1−(0.231−0.013)/0.231]. This estimate is statistically significant at the 5% level.

**Fig. 3.**
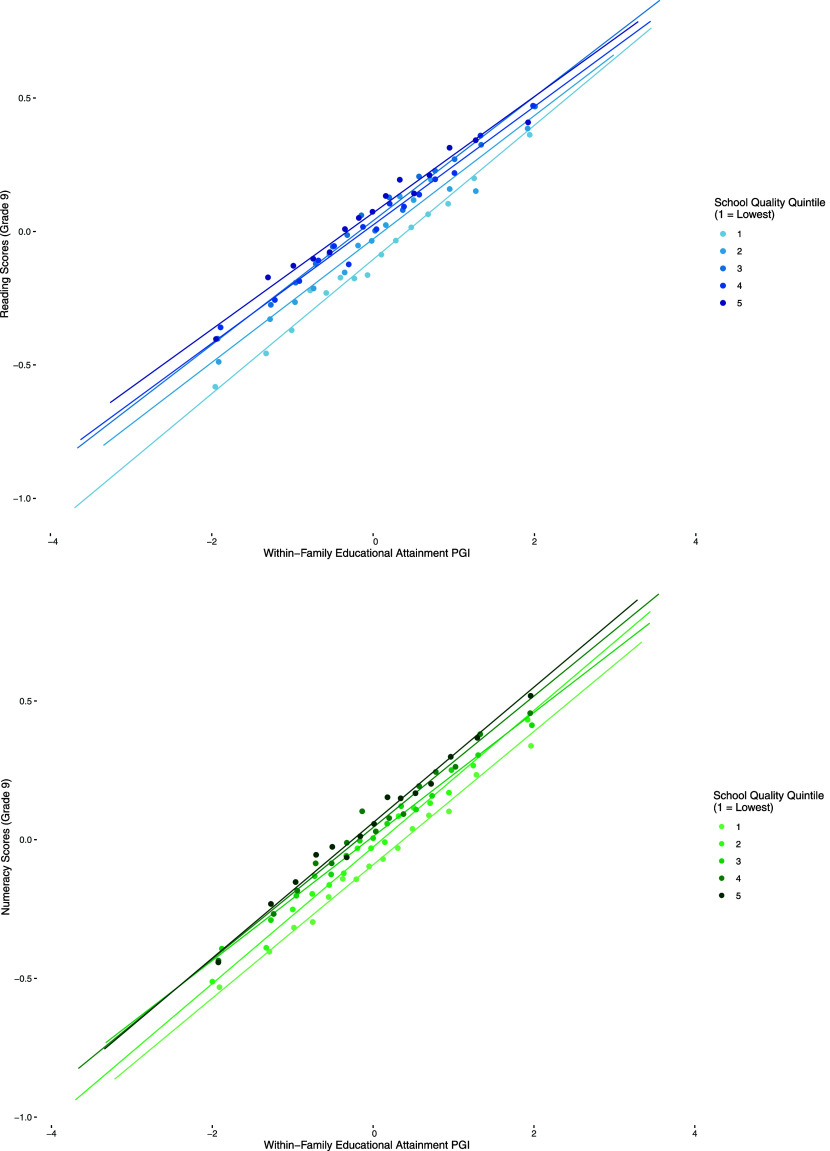
Gene–environment interactions for reading and numeracy test scores. This figure shows binned scatter plots for the relationship between *PGI^EA^* and test scores in grade 9 by quintile of the outcome-specific *VA^d^* distribution. The *Top* panel shows results for reading test scores; the *Bottom* panel shows results for numeracy test scores. Scatter plots are constructed by grouping the *PGI^EA^* distribution into 15 bins. Regression slopes are estimated on the full data, conditioning on controls matching the focal models (see column 3, Table 2 for reading and numeracy; *Materials and Methods*). Data: Own calculations based on MoBa and Norwegian registers.

Theoretically, *PGI^EA^* and *VA^d^* could be complements or substitutes for student learning. If they were complements, school quality (*VA^d^*) would magnify advantages based on *PGI^EA^*; if they were substitutes, *VA^d^* would compensate for disadvantages based on *PGI^EA^*. Our results point to the substitutability of *PGI^EA^* and *VA^d^* as input factors for students’ reading test scores. Fig. 3 illustrates genetic gradients across Norwegian schools of varying quality, revealing whether this substitutability stems from gains at the bottom or losses at the top of the *PGI^EA^* distribution. The genetic gradients are flatter in higher-quality schools. This pattern suggests that in higher-quality schools, genetic differences between children matter less because schools compensate children with lower *PGI^EA^*. Reversely, the impact of genetic differences among students on their test scores is more pronounced in lower-quality schools.

#### Numeracy.

We repeat the previous analysis with numeracy test scores as the outcome of interest. Fig. 3 and *SI Appendix*, Table S1 suggests that there is no gene–environment interaction for numeracy scores. In our preferred specification, a 1 SD increase in *PGI^EA^* is associated with 0.239 SD higher numeracy scores, while a 1 SD increase in *VA^d^* is associated with 0.039 SD higher numeracy scores. The point estimate for the gene–environment interaction is 0.000, with an associated 95% confidence band of [−0.0078, 0.0078]. Therefore, this null finding is precise enough to exclude magnitudes that are approximately half the size of the point estimate for the gene–environment interaction in the reading domain (0.013).

## Discussion

### Summary.

We investigated whether schools can mitigate birth-related educational inequalities by integrating exogenous school VA measures with the natural lottery of within-family genetic variation. Using this stringent causal inference design, we found compelling evidence of a gene–environment interaction influencing reading skill development (though not numeracy) even within the narrow time window of one school year (grade 8, ages 13 to 14). Our results suggest that investments in school quality can promote equitable skills development by effectively narrowing gaps in reading test scores between students with different genetic predispositions. Notably, these findings also reveal a double disadvantage: The skill development gap between children in low versus high-quality schools is even larger for those with less genetic predisposition to education.

### Reading versus Numeracy.

We estimate that increases in school quality reduce the impact of *PGI^EA^* on reading test scores; however, we do not find a gene–environment interaction for numeracy test scores. This result is likely related to the higher persistence of numeracy skills during this developmental period. Examining our preferred specification (column 3 of Table 2) gives an indication of the persistence of skills. In this specification, we control for subject-specific lagged test scores and list the coefficient of this control at the bottom of the table (ρ). If ρ = 0, past achievement does not impact current performance, giving room for new inputs to shape outcomes. Reversely, if ρ = 1, skills are highly persistent, suggesting that new inputs have less scope to shape children’s skills. The corresponding coefficients are 0.462 (*SE* 0.006) for reading and 0.702 (*SE* 0.004) for numeracy test scores. These estimates suggest that numeracy test scores of adolescents in Norway are significantly more persistent than reading test scores, giving high-quality schools less scope to level up the numeracy skills of children with lower *PGI^EA^* relative to their high-*PGI^EA^* peers.

### Magnitude of Effects.

We estimate that a 1 SD increase in school quality reduces the impact of a 1 SD increase of *PGI^EA^* on student outcomes in reading by approximately 6%. To gauge the magnitude of this effect, it is essential to emphasize that this treatment effect captures students’ exposure to high- or low-quality schools for only one school year (grade 8). Lower-secondary education in Norway lasts for 3 y (grades 8-10), with students usually staying in their initial neighborhood school throughout this period [see *SI Appendix*, section A and Kirkebøen (18)]. Therefore, if one were to assume constant and additive treatment effects of *VA^d^* across grades 8-10, a 1 SD increase in school quality would reduce the impact of *PGI^EA^* on student outcomes in reading by approximately 18% over the total duration of lower secondary school. We are aware that the assumption of grade-constant and additive treatment effects is arguably strong and in need of verification by future research. However, it is interesting to note that the implied effect size of this back-of-the-envelope extrapolation is consistent with results from Arold et al. (14), who find that a 1 SD increase in high school quality in the United States (grades 9-12) decreases the impact of *PGI^EA^* on educational attainment by approximately 19%.

### Potential Mechanisms.

The gene–environment interaction identified here can be further understood in the context of theoretical frameworks from economics and developmental psychology. The economics literature on skill formation often conceptualizes student outcomes as a function of students’ initial skills, school inputs, and family inputs, where families adjust their behavior depending on students’ initial skills and school quality (35, 36). Similarly, developmental psychology frameworks propose that development and learning are a product of dynamic interplay between individual biopsychological and social processes (37), where an existing genetic diathesis/vulnerability can be compensated for, controlled, or triggered by proximal social processes (38). Our finding that genetic factors matter less in high-quality schools is consistent with the concept of substitutability from economics, as well as the compensation and diathesis-stress models (13, 38, 39) from developmental psychology, contrasting with bioecological models where social advantage increases genetic expression (37).

The finding that differences in *PGI^EA^* have less impact on the development of reading skills in higher *VA^d^* schools could be explained by both direct and indirect mechanisms. First, students with lower *PGI^EA^* may gain *directly* from attending higher-quality schools. Emerging evidence shows that schools and teachers in industrialized countries focus on the lower parts of the achievement distribution, suggesting that they attach a higher weight to the learning of disadvantaged students (40). Therefore, students with lower *PGI^EA^* who attend better schools receive relatively more and/or higher-quality investments than their peers with higher *PGI^EA^*, which could explain the negative gene–environment interaction in this study. This mechanism assumes that Norwegian educators distribute instructional resources unequally across students within the same school. However, even without this assumption, the negative gene–environment interaction can be explained by diminishing returns to educational inputs. Students with lower *PGI^EA^* may have more room for improvement and, consequently, may gain more from attending a better school. Notably, compensation and triggering are at the ends of a continuum: Just as enriched learning environments may compensate for genetic disadvantage, lower-quality schools could be stressful environments that “trigger” genetic predispositions linked to low educational attainment and hinder the accumulation of reading skills^*^

Second, students with lower *PGI^EA^* may gain *indirectly* from attending high-quality schools through family adjustments to school quality and children’s *PGI^EA^*. These indirect mechanisms are more complex as they combine the effects of different inputs on student learning with the behavioral responses of parents. For example, if families prioritize supporting children with higher *PGI^EA^* and family and school inputs act as substitutes in fostering learning, then the effect of additional family inputs received by high-*PGI^EA^* students becomes weaker in high-*VA^d^* schools. This could contribute to the negative gene–environment interaction we observed. Alternatively, if families decrease their investment with increases in school quality, and family inputs and *PGI^EA^* work as complements in learning, then the effect of decreased family inputs received by students in high-*VA^d^* schools will be less pronounced for low-*PGI^EA^* students. This could also contribute to the negative gene–environment interaction observed in this study.

### Limitations.

Our gene–environment interaction findings are specific to genetic variants captured by *PGI^EA^*, derived from a genome-wide association study conducted primarily in populations of European ancestries. This represents only a subset of total genetic influences on reading ability. Therefore, while we demonstrate that school quality moderates genetic effects shared between reading and *PGI^EA^*, we cannot determine whether all genetic influences on reading are similarly affected by school environments. Moreover, the notion that within-family polygenic index associations can be interpreted causally has been subject to debate; a key caveat being that within-family associations are causal for families heterozygous at relevant variants but may not generalize (41, 42). Additionally, while the standardized outcomes we analyzed were not skewed, the raw test scores showed negative skew that may indicate measurement limitations at the upper end of the scale, and we cannot rule out that this could contribute to the interaction effects we observe.

Furthermore, we emphasize that our results are context dependent. High-*VA^d^* schools can increase average student outcomes by different policies that focus on low-ability students, high-ability students, or broad policies that are equally effective for all students regardless of their ability (43, 44). These practices may vary across countries, grades, subjects, and schools. We find that lower secondary schools in Norway with high-*VA^d^* increase the reading scores of all students, but with higher relative gains of low-*PGI^EA^* students (Fig. 3). The resulting negative interaction of *PGI^EA^* and *VA^d^* may be attenuated or even reversed in contexts where schools put a stronger emphasis on the learning of high-ability students. Such differences may even emerge within the same country, for example, if a school system’s goals shift from equalizing students’ opportunities in lower grades to differentiating students at higher grades. Therefore, we encourage caution when extrapolating our results to other contexts.

### Future Research.

This study forges several interesting avenues for future research. Ideally, investigations aiming to distinguish between different mechanisms involved in the gene–environment interaction that we identified should combine the data prerequisites for causal gene–environment interplay studies with detailed data on school practices and parental inputs. The former will allow us to understand the characteristics of high-quality schools and to study which features of these schools make them particularly beneficial to students with lower *PGI^EA^* (see also our discussion on *direct* gene–environment effects). The latter will allow us to understand how mothers and fathers adapt their parenting strategies in response to their children’s *PGI^EA^* and the quality of their schools, and whether particular parental inputs are especially beneficial to students with lower *PGI^EA^* (see also our discussion on *indirect* gene–environment effects).

The relevant school characteristics and parental inputs are highly multifaceted and are unlikely to be captured in a single dataset. School quality is likely to consist of diverse pedagogical, organizational, cultural, relational, financial, and physical aspects. Similarly, parental inputs may consist of different time and monetary investments as well as parenting styles. However, the increased availability of molecular genetic data and the integration of these data with linked register, survey, and cohort study datasets paves the way for researchers to address these important questions convincingly in the future.

## Materials and Methods

### Data.

#### The Norwegian Mother, Father, and Child Cohort Study (MoBa).

MoBa is a prospective population-based pregnancy cohort study conducted by the Norwegian Institute of Public Health (19). Pregnant women were recruited from across Norway from 1999 to 2009. The women consented to initial participation in 41% of the pregnancies. Of the fathers invited to participate, 83% consented. The total cohort includes approximately 114,500 children, 95,200 mothers, and 75,200 fathers. MoBa participants were linked to administrative register data through the Norwegian national ID number system. Analyses are conducted on MoBa children born 2002-2008 with grade 9 national test scores in reading and numeracy, complete data for genome-wide genotyping [see *SI Appendix*, section C and Corfield et al. (45) for details on genotyping and genetic quality control in MoBa], information on *VA^d^* in their school-cohort cell, and nonmissing control variables (*N* = 30,939).

#### Norwegian register data.

We estimate *VA^d^* for standardized test scores in reading and numeracy in grade 8. Since standardized tests are conducted at the beginning of the academic year, we can use test scores in grades 8 and 9 to measure student progress in grade 8, i.e., the first year of lower secondary school (*SI Appendix*, sections A and B). We construct *VA^d^* using register data on the entire Norwegian student population in birth cohorts 1997-2007 (approximately 60,000 per cohort). The earliest birth cohort that completed comparable standardized tests in grades 8 and 9 in reading and numeracy is 1997. While 2007 was the latest complete birth cohort available during data construction, a small number of children born in 2008 (n = 13) also met all inclusion criteria and were included in the analytical sample.

### Treatment Variables.

#### Polygenic index for educational attainment (*PGI^EA^*).

We used beta weights from the largest genome-wide association study of educational attainment to date (“EA4”), excluding MoBa (46). Polygenic indices were calculated using LDPred v.1 (47), a Bayesian approach that uses a prior on the expected polygenicity of a trait (assumed fraction of nonzero effect markers) and adjusts for linkage disequilibrium (LD) based on a reference panel to compute weights for individual single nucleotide polymorphisms (SNPs). LD adjustment was performed using the MoBa genotype data as LD reference panel. The weights were estimated based on the heritability explained by the markers in the GWA summary statistics and the assumed fraction of markers with nonzero effects. *PGI^EA^* were computed based on these weights with the –score command in plink2 (48).

#### School value-added (VA^d^).

Consider educational outcome *Y* in subject *d* of student *i* attending school *j* in cohort *c*. We model this educational outcome as a function of individual student characteristics *Z* and true school quality *VA^d^*:

[1] $$Y_{\mathrm{ijc}}^{d}=\beta^{d}Z_{\mathrm{ijc}}+{\mathrm{VA}}_{\mathrm{jc}}^{d}+\varepsilon_{\mathrm{ijc}}^{d}.$$

In our setting, *Z* comprises lagged grade 8 test scores in numeracy, reading, English, maternal and paternal years of education, second-generation migration status, gender, birth cohort, birth order, number of siblings, and school-cohort averages of all previous controls. See also *SI Appendix*, Table S1 where we provide an overview of all control variables used for the estimation of *VA^d^* and in our main analysis. Note that the specification of *Z* goes beyond lagged test scores in the outcome of interest by controlling for lagged test scores in three subjects, as well as the corresponding aggregates at the school-cohort level. This practice is motivated by methodological research showing the necessity of additional measures of prior achievement for unbiased *VA^d^* estimates (49, 50). These papers also suggest that additional controls for socioeconomic background are usually unnecessary after accounting for such a rich set of controls for prior achievement. We nevertheless include these controls in *Z* to err on the side of caution.

Note that true *VA^d^* is a latent variable captured in the composite error term $\mu_{\mathrm{ijc}}^{d}={\mathrm{VA}}_{\mathrm{jc}}^{d}+\varepsilon_{\mathrm{ijc}}^{d}$ of Eq. **1**. We can construct an estimate of *VA^d^* of school *j* in cohort *c* by estimating Eq. **1** and calculating the cohort-school average in the resulting residuals:

[2] $${\hat{VA}}_{jc}^{d}=\frac{1}{N_{jc}^{d}}\sum_{i}\left(\mu_{ijc}^{d}\right),$$

where $N_{\mathrm{jc}}^{d}$ captures the number of students of cohort *c* in school *j*.

We want to use estimates of *VA^d^* in regression models to explain student outcomes. However, we cannot explain student outcomes of school *j* in cohort *c* using *VA^d^* estimates for the same school cohort because of the mechanical relationship between the dependent variable *Y* and the *VA^d^* estimate (Eq. **1**). For example, a student with a high reading test score will simultaneously push up the corresponding estimate of *VA^d^* in their school-cohort cell. This mechanical link is particularly pronounced if school-cohort cells are small. To break this mechanical relationship, we predict school quality in school *j* of cohort *c* from all neighboring cohorts using an empirical Bayes procedure [see Walters (51) for a recent overview article]:

[3] $$\begin{array}{c} {\underline{VA}}_{jc}^{d}=\sum_{1\leq c^{\prime }\leq C,\;c^{\prime }\neq c}\zeta_{jc^{\prime }}^{d}\;\left({\hat{VA}}_{jc^{\prime }}^{d}\right), \end{array}$$

where ζ are weights selected to minimize forecast errors. Similar to Chetty et al. (26), we use all neighboring cohorts and not just preceding cohorts to increase the precision of the estimates. Therefore, our final estimate of *VA^d^* is the best linear predictor of school quality for cohort *c* in school *j* from all preceding and subsequent cohorts who attended this school while excluding the cohort itself to avoid biased estimates through reversed causality. This procedure yields a noisy estimate of *VA^d^*. Furthermore, it is well known that measurement error in the independent variables leads to attenuation bias in the relevant coefficients in downstream analyses. The empirical Bayes procedure takes care of this concern. Specifically, it chooses weights ζ such that noisy estimates are shrunk to the mean in proportion to their signal-to-noise ratio. It can be shown analytically that this weighting is the exact inverse of attenuation bias in error-in-variables regressions—see Walters (51) for an outline of the formal argument. Therefore, while we estimate *VA^d^* with error, our regressions recover estimates of school effects that are not afflicted by attenuation bias. We note that this conclusion only holds when standardizing *VA^d^* with respect to its true SD, which is unobserved. Therefore, we estimate the true SD by the square root of the 1-y lag autocovariance, which provides a lower bound on the true within-year SD of *VA^d^* (26, 52). We use this estimate for all standardizations of *VA^d^.* In *SI Appendix*, Tables S2 and S3, we also present robustness analyses based on the observed SD, which provide an overestimate of the true within-year SD of *VA^d^*.

In all our analyses, we use $\underline{VA}$*^d^* from Eq. **3** as our estimate of *VA^d^*. However, to simplify the notation, we omit the overbar and refer to this estimate as *VA^d^* in the following. We estimate *VA^d^* using the *vam* command (53) in Stata 18.0.

The outlined procedure yields an unbiased estimate of *VA^d^* if there is no selection into schools based on factors not captured in observable characteristics *Z*. Following Chetty et al. (26), we can evaluate the plausibility of the exogeneity assumption using “as-if-unobservable” variables. Specifically, we treat students’ grade 5 reading and numeracy scores, fathers’ and mothers’ earnings rank at age 6, and fathers’ and mothers’ age at birth as unobserved variables that we do not include in the control vector *Z*. In turn, we can test whether they confound the relationship between *VA^d^* and student outcomes.

The validation exercise consists of three steps and is performed in Stata 18.0 using the *regress* command. First, we separately regress each of our outcomes of interest and the as-if-unobservables on *Z* and store the residuals. This step ensures that we only exploit variation that is not captured by Z. Second, we regress each (residualized) outcome on all (residualized) “as-if-unobservables” and store the predicted outcomes. This step creates a summary statistic for variation in the (residualized) outcomes that is accounted for by our (residualized) “as-if-unobservables.” It captures variation in the outcomes of interest that is not accounted for by *Z* and which, therefore, is a potential source for omitted variable bias. Finally, we regress this summary statistic on *VA^d^*. If *VA^d^* is substantially associated with the predicted outcomes, then this indicates that there is selection into schools based on “as-if-unobservables” (i.e., grade 5 test scores, parental earnings rank, and parental age at birth). The results are shown in Fig. 2.

While the results of this validation exercise support the satisfaction of the “selection-on-observables” assumption in our setting, we acknowledge that there remains the risk of confounding through some other unobservable variable that is correlated with student outcomes and school quality but not captured in *Z*. However, existing methodological literature generally shows that observational VA measures like the ones used in this paper by-and-large concur with VA measures using (quasi)-experimental variation once they account for lagged student test scores in *Z* (17, 18).^†^

*VA^d^* can be interpreted as a summary statistic for all school factors contributing to students’ academic progress in skill dimension *d*. While *VA^d^* captures persistent differences in quality across schools, it does not capture within-school differences in quality due to, for instance, teacher quality.

### Analysis.

We estimate the following model through ordinary least-squares and cluster SE at the level of schools *j*, using *lm_robust* in the estimatr R package (R version 4.2):

[4] $$Y_{\mathrm{ijc}}^{d}=\alpha^{d}{\mathrm{PGI}}_{\mathrm{ijc}}^{\mathrm{EA}}+\beta^{d}{\mathrm{VA}}_{\mathrm{jc}}^{d}+\kappa^{d}\;(PGI_{\mathrm{ijc}}^{\mathrm{EA}}\times {\mathrm{VA}}_{\mathrm{jc}}^{d})+\delta^{d}X_{\mathrm{ijc}}+\varepsilon_{\mathrm{ijc}}^{d}.$$

*PGI^EA^* and *VA^d^* are the variables of interest, *X* is a vector of control variables, and ε^d^ is the error term. α^d^, β^d^, and κ^d^ are the parameters of interest, identifying the causal effects of *PGI^EA^, VA^d^*, and the corresponding gene–environment interaction.

Controls *X* include genetic controls, i.e., paternal and maternal *PGI^EA^*, and genotyping batch, and the vector of covariates *Z* used for the construction of *VA^d^*, i.e., lagged grade 8 test scores in reading, numeracy, English, maternal and paternal years of education, second generation migration status, gender, birth cohort, birth order, number of siblings, and school-cohort averages of all previous controls (*SI Appendix*, Table S1). Note that lagged test scores are a function of *PGI^EA^*. Therefore, they are “bad controls” for estimating genetic effects (34). To address this concern, we regress grade 8 test scores in reading, numeracy, and English on *PGI^EA^* and include the residuals from these regressions as our controls for lagged test scores. Hence, we control for all variations in lagged test scores uncorrelated with our variable of interest (*PGI^EA^*).

## Ethics

The establishment of MoBa and initial data collection was based on a license from the Norwegian Data Protection Agency and approval from the Regional Committees for Medical and Health Research Ethics. The MoBa cohort is now based on regulations related to the Norwegian Health Registry Act. The current study was approved by the Regional Committees for Medical and Health Research Ethics (#2017/2205).

## Supplementary Material

Appendix 01 (PDF)

## Data Availability

Some study data available. Instructions for access to MoBa data from the Norwegian Institute of Public Health can be found here: https://www.fhi.no/en/studies/moba/for-forskere-artikler/research-and-data-access/ (54).

## Footnotes

^*^Theoretically, the existence of this effect is ambiguous. Extensive literature has emphasized the importance of dynamic complementarities in skill formation, which suggests higher gains by students with higher initial skills (55). However, consistent with our results, various recent papers have suggested that higher quality schools have more positive effects on students from disadvantaged socioeconomic backgrounds and who have higher genetic predisposition to ADHD and low educational attainment (13, 14, 16). Other studies have obtained inconclusive or null findings (11, 12). Some of these inconsistencies could be explained by differences in outcome selectivity (i.e., national tests versus degree completion) and/or differences across institutional contexts (56).

^†^Our discussion focuses on the “selection-on-observables” assumption as the key identifying assumption. Reardon and Raudenbush (57) discuss a set of additional identifying assumptions, including i) manipulability; ii) no interference between units; iii) interval scale metric; iv) homogeneity of effects; and v) functional form. Their simulations show that especially departures from iv) may affect VA estimates. The relaxation of iv), however, brings its own econometric challenges in the form of contamination biases (58). Against this backdrop, Angrist et al. (17) highlight the need for further methodological research to address this concern in the future.
