# Supplementary material for: The genetic lottery goes to school: Better schools compensate for the effects of students’ genetic differences
Source: Proc Natl Acad Sci U S A. 2025 Oct 24;122(43):e2511715122. doi: 10.1073/pnas.2511715122 (PMC12582282; doi:10.1073/pnas.2511715122)
Supplement: Supplementary file 1 — Appendix 01 (PDF) [file pnas.2511715122.sapp.pdf]

Supporting Information for

The genetic lottery goes to school: Better schools compensate for the effects of students' genetic differences

Rosa Cheesman[1,2*], Nicolai T. Borgen[3], Astrid M. J. Sandsør[3], and Paul Hufe[4]

1. Department of Psychology, University of Oslo, P.O. box 1094, Blindern, 0317 Oslo, Norway.

2. PsychGen Center, Norwegian Institute of Public Health, Oslo, Norway.

3. Center for Research on Equality in Education (CREATE) and Department of Special Needs Education, University of Oslo, P.O. box 1140 Blindern, 0318, Oslo, Norway.

4. School of Economics, University of Bristol, Bristol BS8 1TU, United Kingdom.

*Correspondence to Rosa Cheesman (r.c.g.cheesman@psykologi.uio.no)

This PDF file includes:

A. Norwegian school system

B. National standardized tests

C. Genotyping

D. Register data measures

E. Supplementary Figures

F. Supplementary Tables

SI References

A. Norwegian school system

The Norwegian compulsory school system consists of elementary education (grades 1-7) and lower secondary education (grades 8-10). Nearly all children attend their public neighborhood school, where children are automatically enrolled. Parents can apply to attend a different public school, but this is only possible if there are spaces available, and enrollment is then determined by the principal on a case-by-case basis. Only about 5% of students attend private schools that are either religious, offer alternative pedagogies, or are international. Public schools are free of charge and have a common national curriculum. There is no tracking, and grade promotion or retention is very rare. Most children have the same classmates while attending the same school across grade levels, and teachers will often follow the same students across grade levels as well. Children do not receive grades during elementary school. During lower secondary school, children receive teacher-assessed grades biannually in each subject. Only the last semester's grades in grade 10 are part of the final transcript and are used, together with results on external exams, to apply to upper secondary education. Students are guaranteed a spot in upper secondary education in one of their three preferred programs (5 academic programs and 10 vocational programs), but the final transcript determines program admission and also school admission if the counties have free school choice within the program.

Norway is recognized as an egalitarian society with lower levels of inequality compared to many other Western countries. This relative equality can be attributed to Norway's low levels of income inequality, comprehensive redistributive welfare state institutions, and high rates of intergenerational mobility. In line with other Nordic countries, Norway exhibits smaller between-school differences (1), likely due to less sorting across schools as well as potentially fewer differences in school quality. Schools serving disadvantaged students are often allocated more resources, such as a higher teacher-student ratio in lower-performing schools (2). However, despite being relatively egalitarian, one in ten children grows up in families with persistently low household income (3). Socioeconomic achievement gaps in Norway are narrower than in countries such as the United States, yet the difference is smaller than expected given substantial cultural and economic differences between the countries (4-6).

B. Standardized national tests

National tests in reading, numeracy, and English are taken annually at the beginning of grades 5 and 8, while numeracy and reading are also tested in grade 9. The grade 9 test is the same as that in grade 8 and measures growth during the first year of lower secondary education. In each academic year, the grade 8 to 9 tests are identical; however, for the individual child, items will vary across grades 8 and 9 as they take these tests in two different academic years. The content of the test is comparable across years.

In the present study, we used numeracy and reading tests for grade 9 as our main outcome variables, where we standardized the summed test scores with a mean of 0 and a SD of 1 within each test and year. Alternative approaches to vertical scaling are conceivable, but we note that there is currently no scientific consensus on the best approach to vertical scaling (7). Reassuringly, methodological comparisons suggest that value-added estimates for teachers and schools are highly correlated across different approaches to vertical scaling (8). Therefore, we do not expect strong effects of alternative scaling choices on our results.

The tests are commissioned from experts in test development and psychometrics connected to universities in Norway. The tests are digital, and the results are scored automatically. About 95% of all students in Norway take the tests, although students with special needs and those following introductory language courses may be exempt. The test results are mainly used to collect information about students' skills and to track school development over time. Results

are conveyed to teachers, students, and parents, but have no direct consequences for students. National tests in grade 8 are used as a control variable, while the ones in grade 5 are used to validate the design.

C. Genotyping

Blood samples were obtained from both parents during pregnancy and from mothers and children (umbilical cord) at birth. Quality-controlled genotyping array data is available for the full 207,569 unique MoBa participants (9). Phasing and imputation were performed with IMPUTE4.1.2_r300.3, using the publicly available Haplotype Reference Consortium release 1.1 panel as a reference. To identify a sub-population of European-associated ancestry, principal component analysis (PCA) was performed with 1000 Genomes phase 1 after LD-pruning. During post-imputation quality control, the following thresholds were used for SNP removal: imputation quality (INFO) score $\leq$ 0.8; MAF<1%; call rate<95%.

D. Register data measures

Stata 18.0 is used to construct all the measures based on register data.

Test scores in reading and numeracy in grades 5, 8, and 9 and English in grades 5 and 8 were constructed using standardized national tests (*prove*) from 2010 to 2021 from the National Education Database (NUDB). A few individuals have been enrolled in multiple tests, due to, for example, switching schools around the timing of the test. As we cannot observe the exact day of testing, we have kept information on the test with the highest score for these few individuals (1,773 individual tests out of 5,826,408 tests are deleted). We keep information on individuals who are registered as having participated in the test (*deltattstatus* equals D), which is 95.09% of the sample in our test cohorts. Test scores are then standardized to have a mean of 0 and a standard deviation of 1 within each year and test.

The school identifier (*orgnr*), used to assign students to schools, is obtained from NUDB and is based on the school where individuals take their national tests in grade 8. As described above, in the few cases where individuals are enrolled in multiple tests in different schools, we keep the school record with the highest score.

Maternal and paternal years of education are measured by translating completed educational degrees (*bu*) the year the child turns six years old in the NUS2000 classification in NUDB into years of schooling with the following categories: primary education (7 years), lower secondary education (10 years), some upper secondary education (12 years), upper secondary education (13 years), bachelor's degree (16 years), master's degree (18 years), and Ph.D. (21 years).

Maternal and paternal earnings ranks are measured by using the sum of employee income and net income from self-employment (*wyrkinnt*) earned during the calendar year the child turns six years old, and is obtained from the Income Register. We rank mothers' income relative to other mothers in the same birth cohort in the year the child turns six, using percentile ranks. Likewise, we rank fathers' income relative to other fathers in the same birth cohort in the year the child turns six. Mothers and fathers with identical values (ties) are given the same rank.

Immigrant background is classified based on the birth country of the child and their parents. We group students into three groups based on the information in the Population Registry.

First, we define native majority children as children born in Norway to two Norwegian-born parents (*invkat* equals A) and children born abroad to two Norwegian-born parents (*invkat* equals G). Second, we define first-generation immigrant children as children born abroad of at least one non-Norwegian-born parent (*invkat* equals B or E). Third, we define second-generation immigrants as children born in Norway of at least one non-Norwegian-born parent (*invkat* equals C or F). In the construction of the school value-added measure, we distinguish between these three groups. When estimating the gene-environment interaction, we restrict the sample to individuals who are born in Norway and who have European-associated genetic ancestry (to avoid population stratification issues). In these analyses, we control for second-generation immigrant status at the individual level and proportion of first-generation immigrants and proportion of second-generation immigrants at the school level.

Several variables are obtained from the Population Registry with minimal or no modifications. Number of siblings (*antsoske*) records the child's total number of siblings, including both full and half-siblings, regardless of their age. Birth order (*paritet*) measures a child's position in the sequence of births among the mother's children. The age of immigration (*innalder*) is defined as the age at which children migrate to Norway. Gender (*kjoenn*) is based on the legal gender as registered in the Norwegian Population Registry and distinguishes between male and female only. Year of birth is obtained as the first four digits of the birth year and month variable (*foedsels_aar_mnd)*. Maternal and paternal age at birth are defined as the difference between the year of birth of the child and the birth years of the mother (*fodtaar_mor*) and father (*fodtaar_far*), respectively. Maternal and paternal identification numbers (*far_lnr* and *mor_lnr*), used to create maternal and paternal earnings rank and education, are based on legal parents in the Population Registry, and are typically biological parents.

E. Supplementary Figures

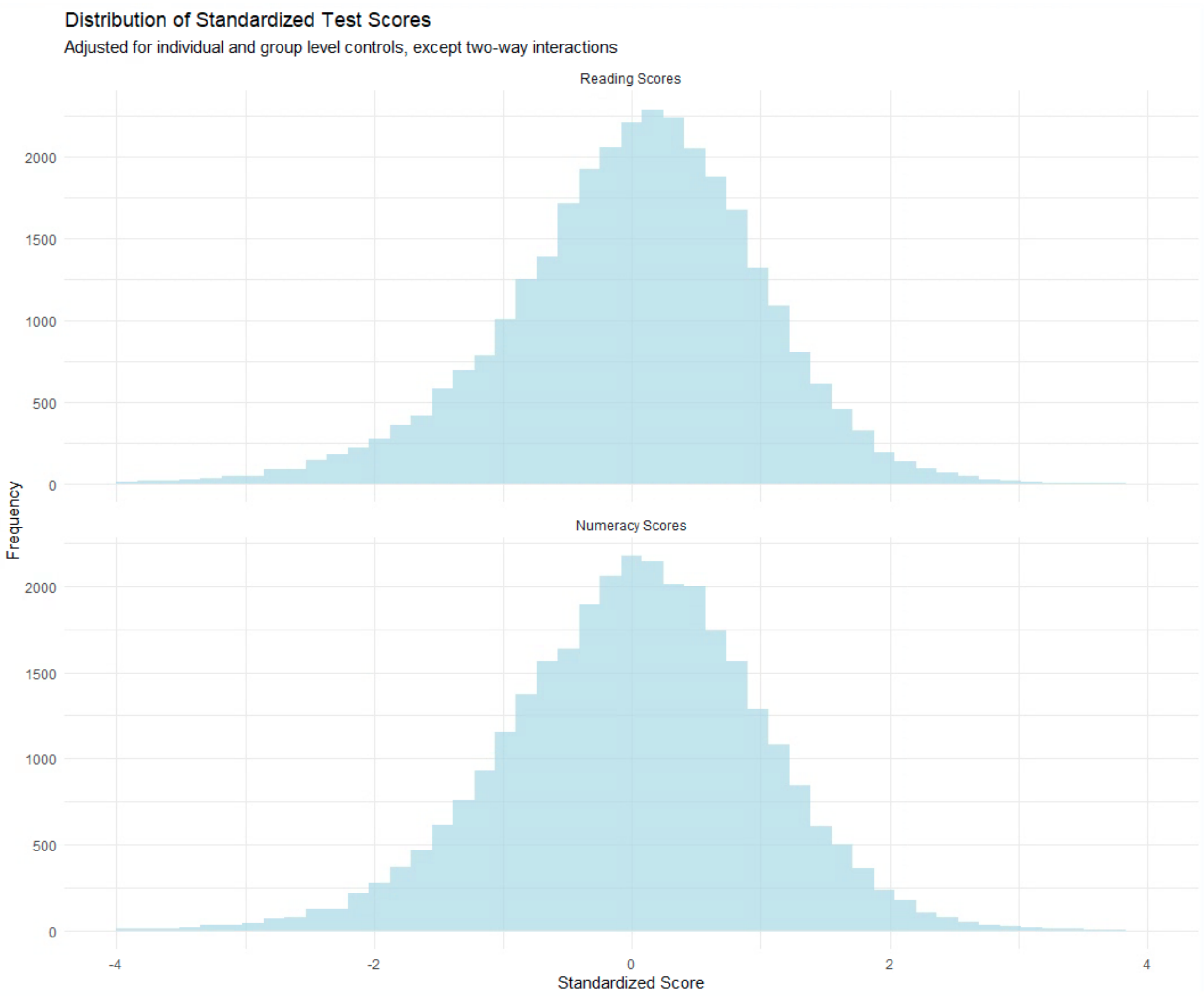

Fig. S1. Test score histograms - assessing ceiling effects and reduced scale sensitivity. Test scores were adjusted for a set of control variables matching our preferred model specification, i.e., column (3) in Table 2 and S.1. If tests have ceiling effects or reduced sensitivity at higher performance levels, this could mechanically create false interactions. Specifically, if high-quality schools produce higher reading scores where the test becomes less sensitive, then genetic effects might appear weaker simply due to measurement limitations rather than true moderation. There is no evidence for ceiling effects. Data: Own calculations based on MoBa and Norwegian registers.

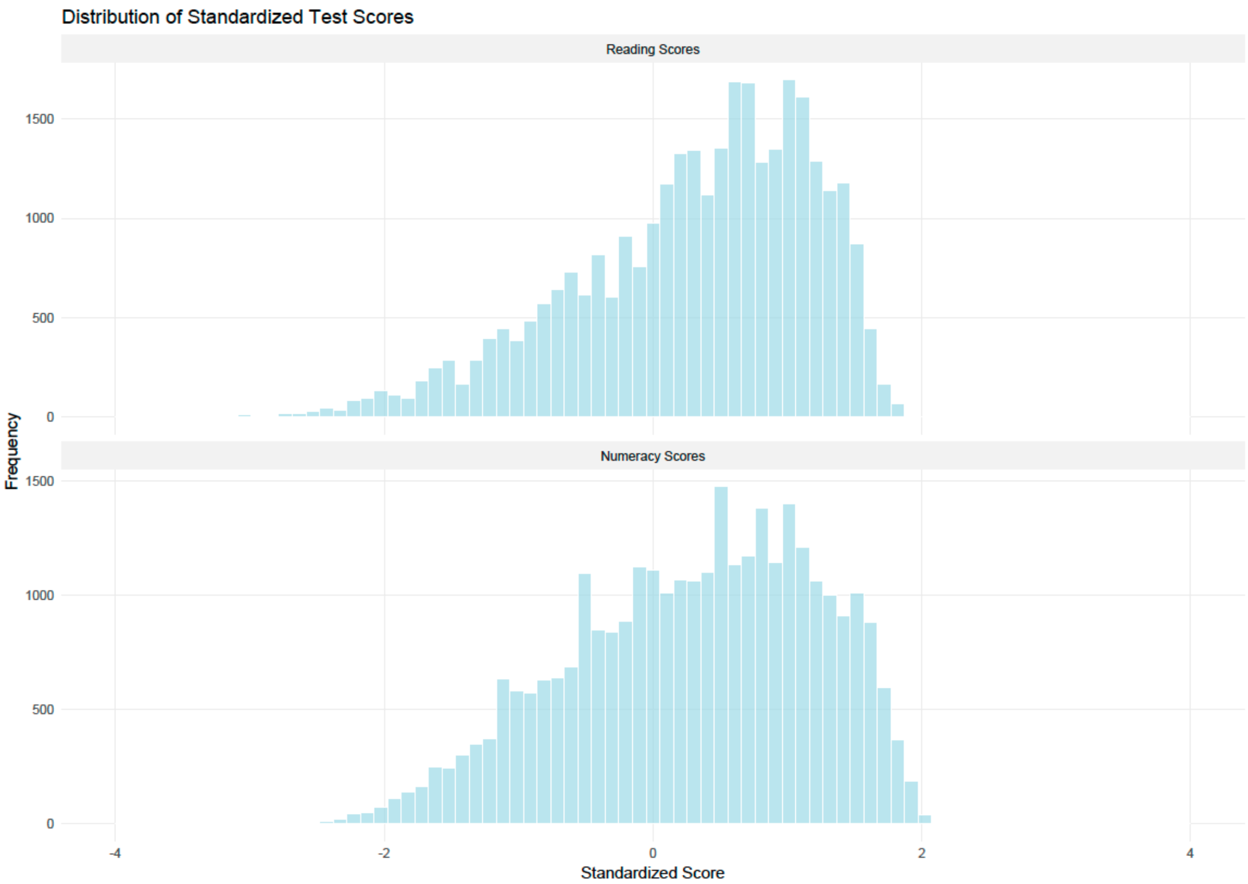

Fig. S2. Raw test score histograms. The raw scores show some negative skew in their distribution. While there is no strong bunching at the maximum values, the skewed distribution may indicate insufficient resolution at the upper end of the scale to adequately discriminate between high-performing individuals. This could potentially compress the upper end of the scale and affect assumed interval properties, which may contribute to the interaction effects observed in our analyses. **Data:** Own calculations based on MoBa and Norwegian registers.

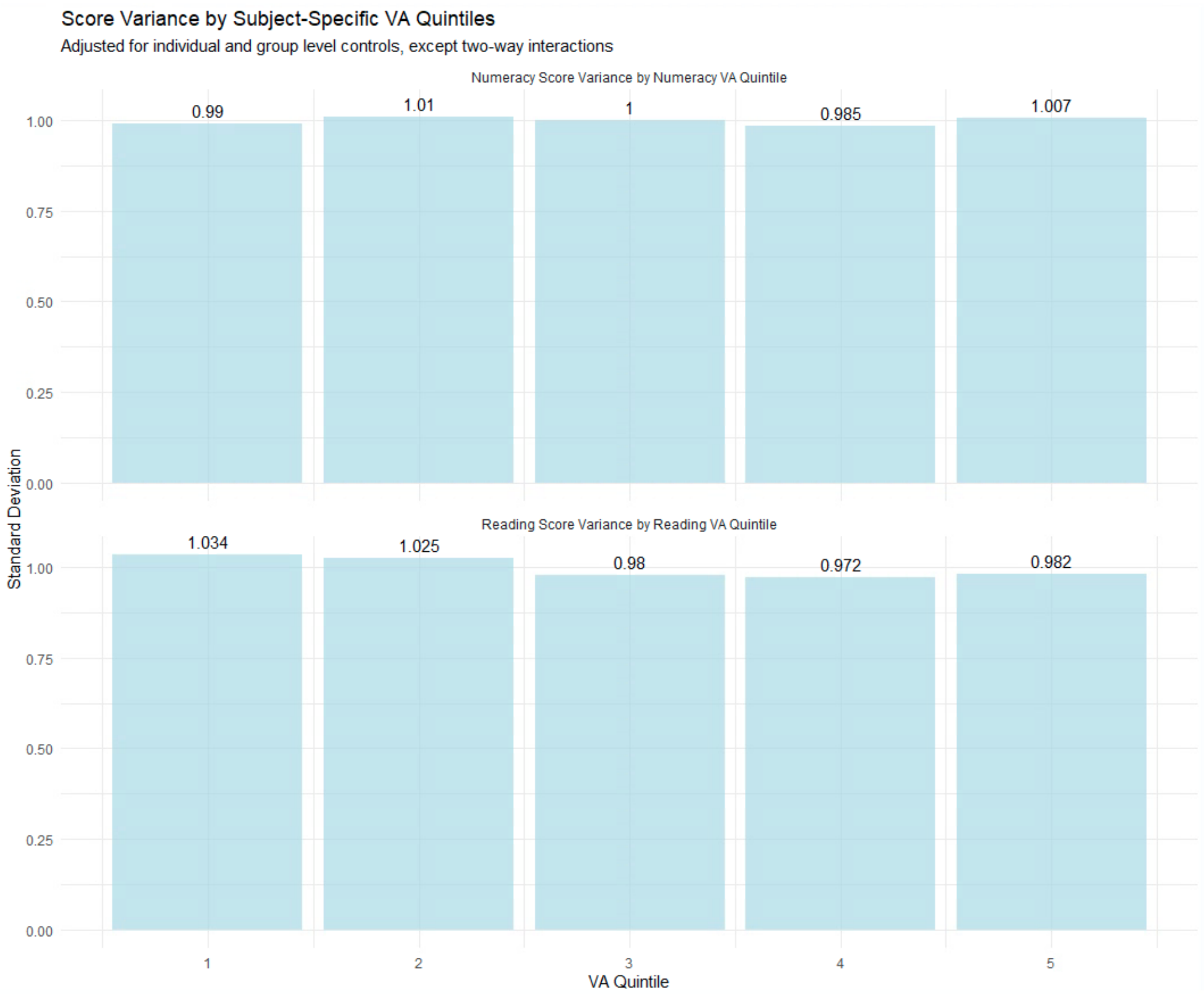

Fig. S3. Test score variability across school quality - assessing dispersion effects. Before calculating variances, test scores were adjusted for a set of control variables matching our preferred model specification, i.e., column (3) in Table 2 and S.1. If better schools simply produce more similar outcomes among students (lower variance), genetic effects might appear weaker due to restricted range (i.e, environmental ‘dimming’ of phenotypic variation) rather than true moderation specific to the genetic and environmental variables under study. The figure shows that schools in the lowest quintile for VA^d in reading have a standard deviation of 1.03 while schools in the top quintile (Q5) have a standard deviation of 0.98. This pattern speaks against spurious gene-environment interactions due to environmental dimming. Data: Own calculations based on MoBa and Norwegian registers.

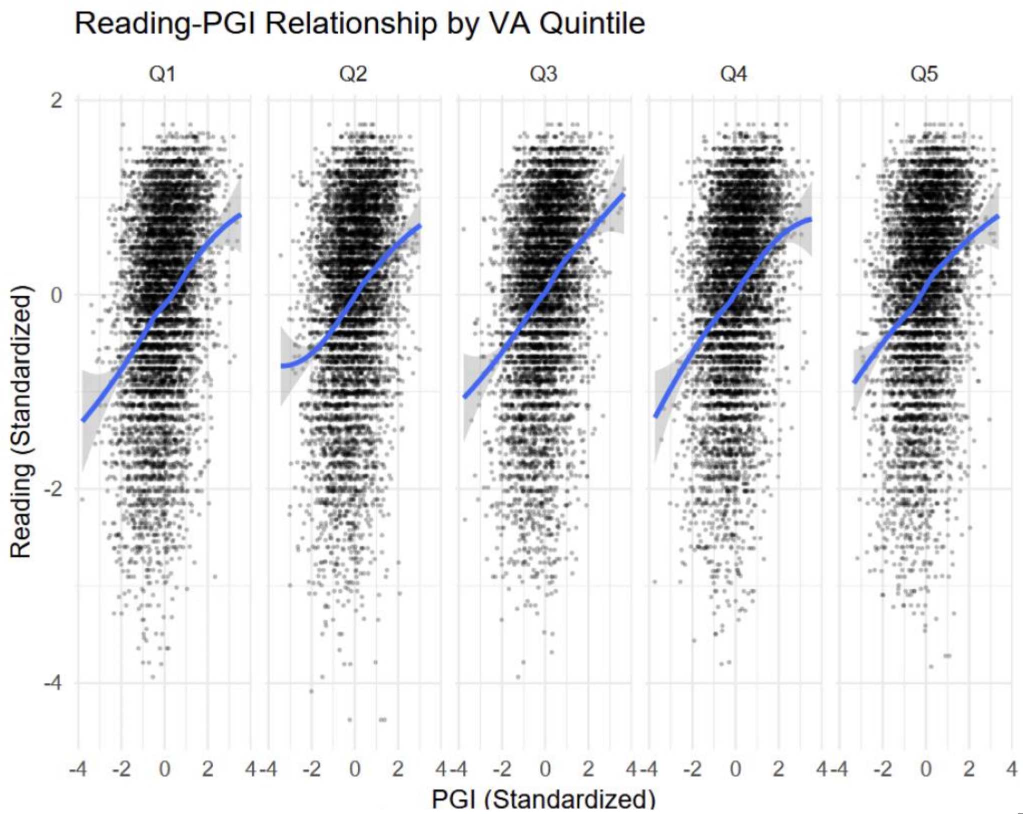

Fig. S4. LOESS (Locally Weighted Smoothing) applied to the reading-PGI^{EA} association, separately for each school quality quintile (raw data). Curved relationships mis-specified as straight lines can create false linear-by-linear interactions between continuous predictors. All quintiles show linear relationships within each school quality level, making it unlikely that non-linearity within groups creates spurious interactions. Each quintile shows a linear relationship, but the slopes decrease from Q1 to Q5. This is consistent with moderation rather than just variance compression. Data: Own calculations based on MoBa and Norwegian registers.

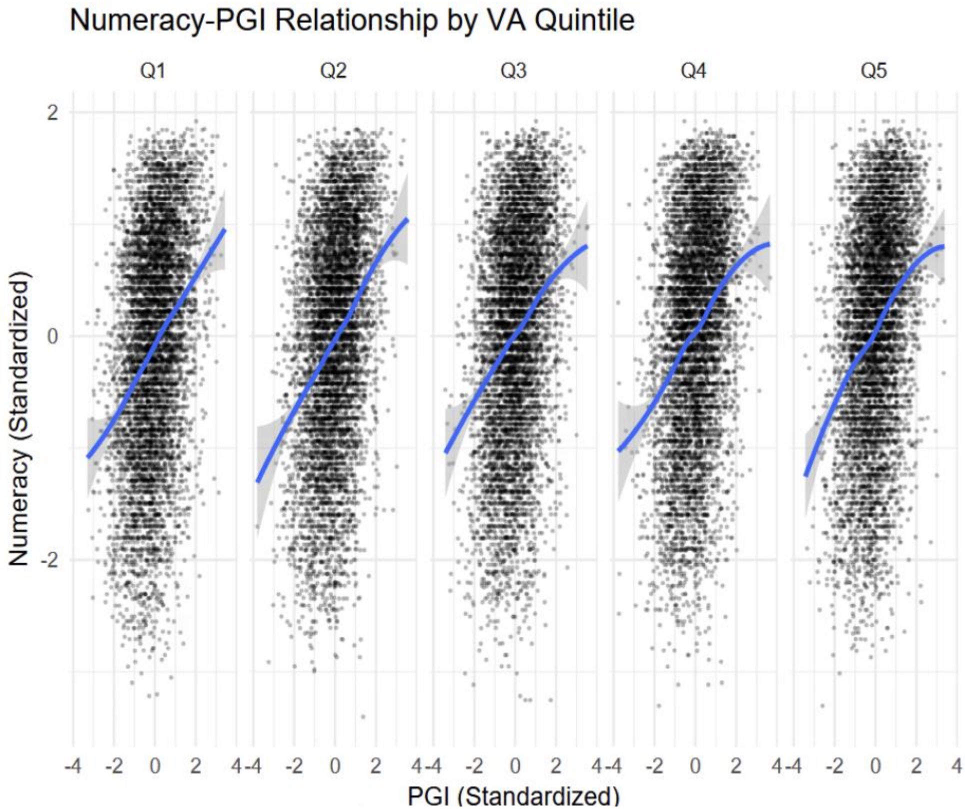

Fig. S5. LOESS (Locally Weighted Smoothing) applied to the numeracy-PGI^{EA} association, separately for each school quality quintile (raw data). Curved relationships mis-specified as straight lines can create false linear-by-linear interactions between continuous predictors. All quintiles show linear relationships within each school quality level, making it unlikely that non-linearity within groups creates spurious interactions. Each quintile shows a linear relationship, and the slopes are similar from Q1 to Q5. Data: Own calculations based on MoBa and Norwegian registers.

F. Supplementary Tables

Covariates for VA^d (Z)	Covariates for main analysis (X)
Panel (a): Individual school selection controls	
Test score (Grade 8, Reading)	Test score (Grade 8, Reading)
Test score (Grade 8, Numeracy)	Test score (Grade 8, Numeracy)
Test score (Grade 8, English)	Test score (Grade 8, English)
Years of education (Mother)	Years of education (Mother)
Years of education (Father)	Years of education (Father)
Second-generation immigrant	Second-generation immigrant
First-generation immigrant	First-generation immigrant*
Age of immigration	Age of immigration*
Birth cohort	Birth cohort
Birth order	Birth order
Number of siblings	Number of siblings
Gender	Gender
Panel (b): Group-level school selection controls (school-cohort average)	
Test score (Grade 8, Reading)	Test score (Grade 8, Reading)
Test score (Grade 8, Numeracy)	Test score (Grade 8, Numeracy)
Test score (Grade 8, English)	Test score (Grade 8, English)
Years of education (Mother)	Years of education (Mother)
Years of education (Father)	Years of education (Father)
Second-generation immigrant	Second-generation immigrant
First-generation immigrant	First-generation immigrant
Age of immigration	Age of immigration
Birth cohort	Birth cohort
Birth order	Birth order
Number of siblings	Number of siblings
Gender	Gender
Panel (c): Genetic controls	
–	PGI^{EA} (Mother)
–	PGI^{EA} (Father)
–	Genotyping batch

Table S1. Overview of control variables. This table shows an overview of the controls used in the estimation of school value-added and our main analysis. Column 1 summarizes the covariate vector Z (equation [1]); Column 2 summarizes the covariate vector X (equation [4]). * indicates variables for which there is no variation in the MoBa genotyped analysis sample as all participants are born in Norway with European-associated ancestries.

Outcome: Reading (Grade 9)	(1)	(2)	(3)	(4)
PGI^{EA}	0.304*** (0.006)	0.230*** (0.008)	0.231*** (0.005)	0.231*** (0.005)
$VA^{Reading}$	0.060*** (0.009)	0.059*** (0.009)	0.034*** (0.005)	0.033*** (0.004)
$PGI^{EA} \times VA^{Reading}$	-0.013* (0.005)	-0.013* (0.005)	-0.009* (0.004)	-0.008 (0.005)
Genetic controls	×	✓	✓	✓
School quality controls	×	×	✓	✓
2-way interactions (PGI^{EA}, VA^d, X)	×	×	×	✓
R^2	0.096	0.104	0.654	0.657
N	30,939	30,939	30,939	30,939
Skill persistence ρ	–	–	0.462*** (0.006)	0.460*** (0.006)

Table S2. Gene-environment interaction for reading scores – robustness to alternative scaling of VA^d. This table shows estimates for the effects of PGI^{EA} and VA^d on children's reading scores in grade 9, as well as the corresponding gene-environment interaction. In contrast to our baseline estimates, we standardize VA^d using the observed SD, not the square root of the one-year lag autocovariance. *Genetic controls* include the PGI^{EA} of biological mothers and fathers, and categorical variables for the genotyping batch. *School quality controls* include lagged grade 8 test scores in reading, numeracy, English, maternal and paternal years of education, second-generation migration status, gender, birth cohort, birth order, number of siblings, and school-cohort averages of all previous controls. *2-way interactions* include all interactions of PGI^{EA} and VA^d with the aforementioned controls. Skill persistence ρ indicates the estimate for lagged test scores in reading (grade 8), which is estimated in the model as part of the child controls. Standard errors (in parentheses) are clustered at the school level. Significance levels: * $p < 0.05$, ** $p < 0.01$, *** $p < 0.001$. Data: Own calculations based on MoBa and Norwegian registers.

Outcome: Numeracy (Grade 9)	(1)	(2)	(3)	(4)
PGI^{EA}	0.314*** (0.006)	0.238*** (0.008)	0.239*** (0.004)	0.239*** (0.004)
$VA^{Numeracy}$	0.056*** (0.010)	0.055*** (0.009)	0.029*** (0.003)	0.029*** (0.003)
$PGI^{EA} \times VA^{Numeracy}$	-0.004 (0.005)	-0.004 (0.005)	-0.000 (0.003)	0.000 (0.004)
Genetic controls	×	✓	✓	✓
School quality controls	×	×	✓	✓
2-way interactions (PGI^{EA}, VA^d, X)	×	×	×	✓
R^2	0.102	0.109	0.738	0.740
N	30,939	30,939	30,939	30,939
Skill persistence ρ	–	–	0.702*** (0.004)	0.703*** (0.004)

Table S3. Gene-environment interaction numeracy scores – robustness to alternative scaling of VA^d. This table shows estimates for the effects of PGI^{EA} and VA^d on children's numeracy scores in grade 9, as well as the corresponding gene-environment interaction. In contrast to our baseline estimates, we standardize VA^d using the observed SD, not the square root of the one-year lag autocovariance. *Genetic controls* include the PGI^{EA} of biological mothers and fathers, and categorical variables for the genotyping batch. *School quality controls* include lagged grade 8 test scores in reading, numeracy, English, maternal and paternal years of education, second-generation migration status, gender, birth cohort, birth order, number of siblings, and school-cohort averages of all previous controls. *2-way interactions* include all interactions of PGI^{EA} and VA^d with the aforementioned controls. Standard errors (in parentheses) are clustered at the school level. Skill persistence ρ indicates the estimate for lagged test scores in numeracy (grade 8), which is estimated in the model as part of the child controls. Significance levels: * $p < 0.05$, ** $p < 0.01$, *** $p < 0.001$. Data: Own calculations based on MoBa and Norwegian registers.

SI References

1. E. Caponera, B. Losito. Context factors and student achievement in the IEA studies: evidence from TIMSS. *Large-scale Assess Educ* **4**, 12 (2016).
2. N.T. Borgen, L. J. Kirkebøen, A. Kotsadam, O. Raaum. Do Funds for More Teachers Improve Student Outcomes? *Journal of Human Resources* (2025)
3. Statistics Norway. Færre barn lever i familier med lavinntekt. [Fewer children live in families with low income.] (2024). https://www.ssb.no/inntekt-og-forbruk/inntekt-og-formue/artikler/faerre-barn-lever-i-familier-med-lavinntekt#:~:text=I%20perioden%202020%2D2022%20tilh%C3%B8rte,sammenlignet%20med%20perioden%202019%2D2021
4. A.K. Chmielewski, S.F. Reardon. Patterns of Cross-National Variation in the Association Between Income and Academic Achievement. *AERA Open*, **2**, (2016).
5. A.K. Chmielewski. The Global Increase in the Socioeconomic Achievement Gap, 1964 to 2015. *American Sociological Review*, **84**, 517-544 (2019).
6. A. M. J. Sandsør, H. D. Zachrisson, L. A. Karoly, E. Dearing.The Widening Achievement Gap Between Rich and Poor in a Nordic Country. *Educational Researcher*, **52**, 195-205 (2023).
7. S.R. Student. Growth on 2019 state achievement tests: Empirical benchmarks and the role of scale choice. *Journal of Research on Educational Effectiveness*, 1–27 (2024).
8. D.C. Briggs, J.P. Weeks. The sensitivity of value-added modeling to the creation of a vertical score scale. *Education Finance and Policy* **4**, 384–414 (2009).
9. E. C. Corfield, et al. The Norwegian Mother, Father, and Child cohort study (MoBa) genotyping data resource: MoBaPsychGen pipeline v.1. BioRxiv (2022) https:/doi.org/10.1101/2022.06.23.496289.
